# Associations between daily physical activity, handgrip strength, muscle mass, physical performance and quality of life in prefrail and frail community-dwelling older adults

**DOI:** 10.1007/s11136-016-1349-8

**Published:** 2016-06-30

**Authors:** Sandra Haider, Eva Luger, Ali Kapan, Sylvia Titze, Christian Lackinger, Karin E. Schindler, Thomas E. Dorner

**Affiliations:** 1 Centre for Public Health, Institute of Social Medicine, Medical University of Vienna, Kinderspitalgasse 15/1, 1090 Vienna, Austria; 2 Institute of Sport Science, University of Graz, Mozartgasse 14/I, 8010 Graz, Austria; 3 Department for Health Promotion and Prevention, SPORTUNION Austria, Falkestraße 1, 1010 Vienna, Austria; 4 Division of Endocrinology and Metabolism, Department of Internal Medicine III, Medical University of Vienna, Waehringer Guertel 18–20, 1090 Vienna, Austria

**Keywords:** Frailty, Quality of life, Muscle mass, Handgrip strength, Balance

## Abstract

**Purpose:**

The aim of this study was to examine the associations between daily physical activity (DPA), handgrip strength, appendicular skeletal muscle mass (ASMM) and physical performance (balance, gait speed, chair stands) with quality of life in prefrail and frail community-dwelling older adults.

**Methods:**

Prefrail and frail individuals were included, as determined by SHARE-FI. Quality of life (QoL) was measured with WHOQOL-BREF and WHOQOL-OLD, DPA with PASE, handgrip strength with a dynamometer, ASMM with bioelectrical impedance analysis and physical performance with the SPPB test. Linear regression models adjusted for sex and age were developed: In model 1, the associations between each independent variable and QoL were assessed separately; in model 2, all the independent variables were included simultaneously.

**Results:**

Eighty-three participants with a mean age of 83 (SD: 8) years were analysed. Model 1: DPA (*ß* = 0.315), handgrip strength (*ß* = 0.292) and balance (*ß* = 0.178) were significantly associated with ‘overall QoL’. Balance was related to the QoL domains of ‘physical health’ (*ß* = 0.371), ‘psychological health’ (*ß* = 0.236), ‘environment’ (*ß* = 0.253), ‘autonomy’ (*ß* = 0.276) and ‘social participation’ (*ß* = 0.518). Gait speed (*ß* = 0.381) and chair stands (*ß* = 0.282) were associated with ‘social participation’ only. ASMM was not related to QoL. Model 2: independent variables explained ‘overall QoL’ (*R* ^2^ = 0.309), ‘physical health’ (*R* ^2^ = 0.200), ‘autonomy’ (*R* ^2^ = 0.247) and ‘social participation’ (*R* ^2^ = 0.356), among which balance was the strongest indicator.

**Conclusion:**

ASMM did not play a role in the QoL context of the prefrail and frail older adults, whereas balance and DPA were relevant. These parameters were particularly associated with ‘social participation’ and ‘autonomy’.

## Background

In community-dwelling older adults, the geriatric syndrome of frailty is common [1]. Frailty is defined as a state of high vulnerability and is caused by malnutrition, chronic inflammation and sarcopenia [2], which is a progressive loss of muscle mass in combination with a decrease in muscle strength or physical performance [3].

The consequences of frailty are adverse health outcomes such as disability, dependency, hospitalisation and need for long-term care [2]. Furthermore, when compared to robust community-dwelling persons, frail adults demonstrate significantly lower quality of life (QoL) [4–7]. Since sufficient energy, freedom from pain and the ability to perform the activities of daily living are important factors influencing QoL [8], it can be assumed that disabilities, physical limitations and deterioration of psychological well-being are possible explanations for the poorer QoL of frail adults [4, 5].

There is evidence that low daily physical activity (DPA) is associated with poor QoL in older adults [9, 10]. Furthermore, previous studies of frail persons have demonstrated that muscle strength, as represented by handgrip strength [3], plays an important role regarding QoL [6, 7, 11]. Since muscle mass is an important prerequisite for muscle strength [12], it is clear that there is also an association between muscle mass and QoL. To the best of our knowledge, no study to date has observed this relationship in prefrail and frail adults. However, some studies have showed that not only loss of muscle mass but also muscle quality (e.g. muscle composition, metabolism, neural activation, fibrosis) contributes to the age-related decline in physical performance and mobility [13–15]. The link between physical performance and QoL in frail adults has been demonstrated in previous research. Accordingly, an association between slowness (assessed by gait speed or the Timed Up and Go test) and QoL has been shown [6, 11]. Furthermore, Gobbens et al. [7] revealed that, in addition to handgrip strength, difficulties in maintaining balance and difficulties in walking are associated with poor QoL in frail adults living in nursing homes.

Since frailty is a public health challenge [16], and the number of frail persons is expected to increase in the future [1], it is of particular importance to better understand the factors associated with poor QoL. Thus, the aim of this analysis was to examine the associations between DPA, handgrip strength, appendicular skeletal muscle mass (ASMM), physical performance and the different QoL domains in prefrail and frail older persons still living in their own homes.

## Methods

### Study sample

Data for this cross-sectional analysis were derived from the baseline assessment of a randomised controlled intervention study, conducted between September 2013 and July 2015 in Vienna, Austria. The study protocol has been previously published [17]. In this study, persons older than 65 years, who were still living in their own homes, were included. These persons had to be prefrail or frail according to the Frailty Instrument for primary care of the Survey of Health, Ageing and Retirement in Europe (SHARE-FI) [18]. SHARE-FI is a sex-specific calculator that includes items concerning exhaustion, weight loss, handgrip strength, slowness and low activity. SHARE-FI is based on discrete factor scores, and it divides persons into robust (female <0.315; male <1.212 points), prefrail (female <2.103; male <3.005 points) and frail (female <6; male <7 points). As prefrail and frail persons were included, females had to score more than 0.315 points and males more than 1.212 points, respectively. In addition, adults at risk of malnutrition or persons who were malnourished according to the Mini Nutritional Assessment Short-Form (MNA^®^-SF ≤ 11 points) were included [19]. As only one participant in the main study was at risk of malnutrition without being at least prefrail, we excluded this person from the present cross-sectional study to harmonise the sample. Furthermore, since the data were baseline data from a randomised trial, participants had to be willing to be visited at home by trained lay volunteers twice a week to perform six strength exercises and talk about nutrition-related aspects [17]. Persons with impaired cognitive function according to the Mini-Mental State Examination (MMSE < 17 points), insufficient German language skills, chemo- or radiotherapy at the moment or planned, insulin-treated diabetes mellitus, chronic obstructive pulmonary disease stage III or IV and patients with chronic kidney insufficiency with protein restriction or on dialysis were excluded. Persons living in nursing homes or retirement housing were also not allowed to participate in the study.

### Measurements

The following measurements were taken at participants’ homes by members of the study team (sports and nutritional scientists). Due to impaired vision, all items of the questionnaires were read aloud to the participants.

#### Daily physical activity (DPA)

The Physical Activity Scale for the Elderly (PASE) [20] was used to assess DPA. This is a validated questionnaire for persons over 55 years [21], which includes items concerning: (1) time spent sitting; (2) time spent walking outdoors; and (3) time spent on light, (4) moderate and (5) strenuous sports [20]. In addition, the following yes or no questions concerning household activity were asked: (6) light household tasks; (7) exhausting household tasks; (8) repair work; (9) light gardening; (10) exhausting gardening; and (11) caregiving activities. In order to analyse the questionnaire, these 11 items were multiplied by a weight score dependent on the level of exhaustion. Finally, all the items were summed. The range of possible scores was from 0 (worst score) to 360 (best score) [20].

#### Appendicular skeletal muscle mass (ASMM)

Body composition was assessed with phase-sensitive bioelectrical impedance analysis (BIA 2000-S device; Data input^®^, Darmstadt, Germany). For this purpose, participants were placed in a supine position and four electrodes were attached to each person’s dominant hand and foot [22]. An alternating current was then passed through the body to measure resistance (*R*) and reactance (*X*c) [23]. ASMM was calculated using the validated formula of Sergi et al. [24]:

$${\text{ASMM }}\left( {\text{kg}} \right) = - 3.964 + \left( {0.227 \times {\text{height}}^{2} /R} \right) + \left( {0.095 \times {\text{weight}}} \right) + \left( {1.384 \times {\text{sex}}} \right) + \left( {0.064 \times X_{c} } \right)$$

#### Handgrip strength

Handgrip strength was measured with a hydraulic dynamometer (Jamar^®^, Lafayette, Louisiana) following the standard procedure [25]. Accordingly, participants were placed in a sitting position on a chair, with their forearms on the arms of the chair and their wrists over the end. The thumb was placed facing upwards. After a short demonstration, each participant performed three attempts on each side, alternating between the right and left hand. Between each attempt, there was a break of 1 min. Finally, the highest value of all six measurements was taken and analysed.

#### Physical performance (balance skills, gait speed, chair stands)

Physical performance was assessed with the Short Physical Performance Battery (SPPB) test [26]. This test is subdivided into three categories, namely, balance skills, gait speed and chair stands. Balance was assessed using side-by-side, semi-tandem and tandem stands. If the first two tasks were possible, participants scored 1 point; if a tandem stand was possible for <3 s, 0 points were given; if a tandem stand was possible for >9 s, participants scored 2 points. Gait speed was tested with a single 4-m walk, with or without assistive devices such as a wheeled walker. Results were divided into four categories (not possible = 0 points; >8.7 s = 1 point; 8.70–6.21 s = 2 points; 6.20–4.82 s = 3 points; <4.82 s = 4 points). The ability to rise from a chair and return to seated position five times with arms crossed was also tested. These results were again divided into four categories (not possible or <60 s = 0 points; >16.7 s = 1 point; 16.69–13.70 s = 2 points; 13.69–11.20 s = 3 points; <11.19 s = 4 points). Finally, a performance score was calculated, summing all the results. The range of possible scores was from 0 (worst) to 12 (best performance).

#### Quality of life (QoL)

The German version of the World Health Organisation Quality of Life-BREF assessment (WHOQOL-BREF) [16], an abbreviated, cross-culturally validated version of WHOQOL-100 [27], was used to assess QoL. The assessment consists of 26 items with a five-point Likert scale response format. The first two questions assess the ‘overall QoL’ of the past 2 weeks, whereas the remaining questions assess QoL in four different domains: ‘physical health’ (seven items), ‘psychological health’ (six items), ‘social relationships’ (three items) and ‘environment’ (eight items). According to the standard procedure [28], all the domains were scored and transformed into a scale ranging from 0 to 100, where a lower value indicates a lower QoL. The ‘social relationships’ domain was calculated using two instead of three items, because only eight participants replied to the question ‘How satisfied are you with your sex life?’

**Fig. 1:**
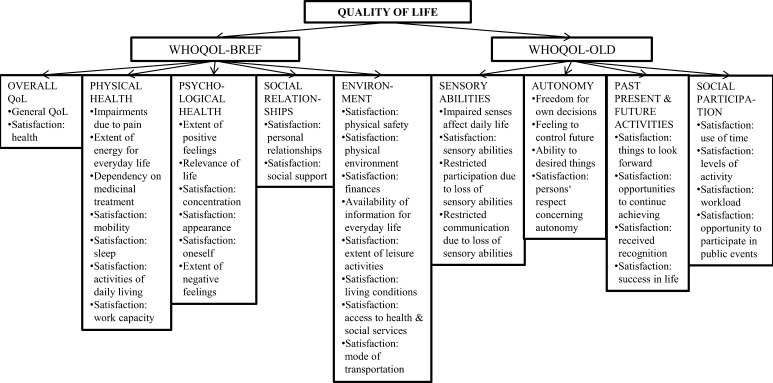
Used domains of WHOQOL-BREF and WHOQOL-OLD and a brief description of their components

In addition to WHOQOL-BREF, the following four domains of the German version of the World Health Organisation Quality of Life-OLD assessment (WHOQOL-OLD) [29] were added: ‘sensory abilities’ (four items), ‘autonomy’ (four items), ‘past, present and future activities’ (four items) and ‘social participation’ (four items). All the QoL domains used in this study and a brief description of their components are shown in Fig. 1.

#### Further measurements

Age, education level (‘elementary school or no degree’, ‘secondary school’, ‘university entrance diploma or higher degree’) and comorbidities were recorded. In addition, each participant’s medication was also documented. Furthermore, body mass index (kg/m^2^) was calculated by dividing the body weight (kg) (as measured by a calibrated scale) by the squared body height (m^2^) (which was measured with a tape).

### Statistical analysis

Based on a median split, study participants were divided into ‘low overall QoL’ (≤ 40 points) and ‘high overall QoL’ (>40 points). Group differences in the continuous variables were assessed by t tests or Mann–Whitney *U* tests, depending on the distribution. For group differences in the categorical variables, Chi-square tests were used, and in the case of the group being smaller than five persons, Fisher’s exact tests were applied. Whenever an item of WHOQOL-BREF or WHOQOL-OLD was missing, the mean of the other items belonging to this domain was calculated [28]. This was undertaken in all domains except for ‘social relationships’, which was calculated with two instead of three items. As a measure of reliability, the internal consistency was determined for each single domain of WHOQOL-BREF and WHOQOL-OLD using Cronbach’s alpha. Furthermore, the correlations between the included independent variables were analysed using Spearman’s or Pearson’s correlation coefficients.

Multiple linear regression analyses were performed to determine the associations between the included variables and QoL. In the first model, a single variable was included as an independent variable, adjusted for age and sex. In the second model, we wanted to identify the strongest indicator for each QoL domain. We also examined how these variables explained each QoL domain. Thus, we undertook a stepwise multiple linear regression analysis including all the variables (DPA, handgrip strength, ASMM, balance skills, gait speed, chair stands). However, we only included variables with a *p* value threshold of 0.20. As in model 1, model 2 was adjusted for sex and age by entering these variables in the models irrespective of their significance. For all the statistical analyses, IBM^®^ SPSS^®^ Statistics 20 software (IBM Corp., Armonk, NY, U.S.) was used. All the tests were two-sided, and a *p* value of <0.05 was considered to be statistically significant.

## Results

In total, 482 people were screened for eligibility, 285 of them in hospitals. Since 208 inpatient individuals close to discharge did not meet the inclusion criteria, 54 refused participation and 19 were excluded for other reasons, only four subjects were recruited in the hospitals. The remaining 80 subjects were recruited via the media: 197 people responded to two editorial features, 47 did not meet the inclusion criteria, 34 refused participation after being provided with detailed project information and 37 were not included for other reasons. Finally, 29 prefrail and 54 frail participants (86 % women) were included in this analysis. Characteristics of the study sample are provided in Table 1.

**Table 1:** Characteristics of the study sample based on a median split of the ‘overall quality of life’ variable

	Total (*n* = 83)	Low overall quality of life (≤40 points) (*n* = 47)	High overall quality of life (>40 points) (*n* = 36)	*p* value
Age (years)	82.6 (8.1)	81.4 (8.4)	84.2 (7.5)	0.115
Sex				
Female	86 %	87 %	84 %	0.617
Male	14 %	13 %	16 %	
Living arrangement				
Alone	75 %	74 %	75 %	0.956
With others	25 %	26 %	25 %	
Education				
Elementary school or no degree	53 %	72 %	53 %	0.150
Secondary school	35 %	40 %	45 %	
University entrance diploma or higher degree	12 %	8 %	22 %	
Frailty status (score)	2.83 (1.1)	3.18 (0.9)	2.36 (1.0)	<0.001
Prefrail	35 %	19 %	56 %	0.001
Frail	65 %	81 %	44 %	
Nutritional status (score)	26.4 (2.8)	25.9 (3.1)	27.1 (2.3)	0.071
Normal nourished	43 %	45 %	61 %	0.032
At risk of malnutrition	33 %	40 %	39 %	
Malnourished	8 %	15 %	0 %	
Body mass index (kg/m^2^)	27.1 (4.5)	27.3 (4.6)	26.9 (4.5)	0.724
Comorbidities				
Cardiac insufficiency	17 %	15 %	28 %	0.149
Peripheral arterial disease	4 %	2 %	6 %	0.576
Hypertension	60 %	74 %	69 %	0.612
Diabetes mellitus type 2	14 %	23 %	6 %	0.020
Chronic rheumatism	7 %	15 %	0 %	0.015
WHOQOL-BREF domains				
Overall quality of life	43.1 (16.5)	32.3 (12.4)	57.2 (8.6)	<0.001
Physical health	47.7 (16.7)	42.3 (14.1)	54.7 (17.4)	0.001
Psychological health	61.6 (16.0)	54.7 (14.3)	70.5 (13.7)	<0.001
Social relationships	74.4 (21.7)	74.2 (22.6)	74.7 (20.8)	0.923
Environment	75.0 (12.3)	71.3 (12.8)	79.9 (8.9)	0.001
WHOQOL-OLD domains				
Sensory abilities	48.0 (22.6)	46.6 (24.0)	49.9 (20.8)	0.517
Autonomy	53.6 (14.9)	49.5 (15.3)	59.1 (12.5)	0.003
Past, present and future activities	54.3 (12.8)	51.4 (12.1)	58.1 (12.9)	0.021
Social participation	43.7 (12.8)	37.8 (11.1)	51.4 (10.7)	<0.001
Physical activity parameters				
Daily physical activity (score)	13.6 (0.0–125.6)	13.6 (0.0–80.0)	28.8 (0.0–125.9)	0.009
Appendicular skeletal muscle mass (kg)	16.9 (3.4)	16.5 (3.3)	17.4 (3.3)	0.274
Handgrip strength (kg)	16.8 (7.2)	15.2 (7.6)	18.9 (6.2)	0.023
Short physical performance battery (score)	4.9 (2.8)	4.3 (2.6)	5.6 (2.9)	0.039
Balance skills (score)	2.0 (1.3)	1.7 (1.2)	2.4 (1.2)	0.008
Gait speed (score)	1.9 (1.0)	1.8 (1.0)	1.9 (1.1)	0.551
Chair stands (score)	1.0 (0.0–4.0)	1.0 (0.0–4.0)	1.0 (0.0–4.0)	0.381

The data are presented in mean (standard deviation) or median (minimum–maximum) or percentages

Group differences: Chi-square test or Fisher’s exact test for categorical data, *t* test or Mann–Whitney *U* test for continuous data

Using Cronbach’s alpha, the internal consistency was determined for each single domain: ‘overall QoL’ (*α* = 0.662), ‘physical health’ (*α* = 0.673), ‘psychological health’ (*α* = 0.658), ‘social relationships’ (*α* = 0.580), ‘environment’ (*α* = 0.624), ‘sensory ability’ (*α* = 0.919), ‘autonomy’ (*α* = 0.640), ‘past, present and future activities’ (*α* = 0.636) and ‘social participation’ (*α* = 0.491).

The correlation coefficients within the included independent variables are shown in Table 2. Accordingly, DPA was found to be associated with balance skills, gait speed and chair stand, but not with handgrip strength and ASMM. The strongest significant correlation was found between DPA and balance skills. Furthermore, a moderate association between handgrip strength and ASMM was identified along with a weak association between handgrip strength and chair stands. In Table 3, the associations between each independent variable and the QoL domains, adjusted for sex and age, are presented. In this regard, DPA was found to be significantly associated with ‘overall QoL’ as well as with ‘physical health’, ‘psychological health’, ‘autonomy’ and ‘social participation’. Handgrip strength was found to be significantly related to ‘overall QoL’. Balance skills was found to be associated with ‘overall QoL’ and the QoL domains of ‘physical health’, ‘psychological health’, ‘environment’, ‘autonomy’ and ‘social participation’. Gait speed and chair stands were found to be related to **‘**social participation’ only.

**Table 2:** Correlations between included independent variables

	Daily physical activity		Handgrip strength		Appendicular skeletal muscle mass		Balance skills		Gait speed		Chair stands	
	*r*	*p* value	*r*	*p* value	*r*	*p* value	*r*	*p* value	*r*	*p* value	*r*	*p* value
Daily physical activity			0.188	0.089	0.213	0.066	**0.498**	**<0.001**	**0.343**	**0.002**	**0.297**	**0.006**
Handgrip strength	0.188	0.089			**0.446**	**<0.001**	0.164	0.138	0.207	0.051	**0.217**	**0.048**
Appendicular skeletal muscle mass	0.213	0.066	**0.446**	**<0.001**			0.138	0.152	−0.071	0.542	0.054	0.648
Balance skills	**0.498**	**<0.001**	0.164	0.138	0.152	0.193			**0.470**	**<0.001**	**0.566**	**<0.001**
Gait speed	**0.343**	**0.002**	0.207	0.061	−0.071	0.542	**0.470**	**<0.001**			**0.503**	**<0.001**
Chair stands	**0.297**	**0.006**	**0.217**	**0.048**	0.054	0.648	**0.566**	**<0.001**	**0.503**	**<0.001**		

*n* = 83

Pearson’s correlation coefficient for normally distributed data and Spearman’s correlation coefficient for data that are not normally distributed

Significant results are shown in bold

**Table 3:** Model—linear regression models including one independent variable (e.g. handgrip strength) and one QoL domain (dependent variable), adjusted for sex and age

	Daily physical activity	Handgrip strength	Appendicular skeletal muscle mass	Balance skills	Gait speed	Chair stands
WHOQOL-BREF						
Overall QoL						
*R* ^2^	0.129	0.272	0.089	0.366	0.151	0.158
Standardised *ß*	**0.315**	**0.292**	0.138	**0.178**	0.068	0.070
*p* value	**0.008**	**0.017**	0.352	**0.001**	0.179	0.160
Physical health						
*R* ^2^	0.114	0.039	0.073	0.162	0.048	0.229
Standardised *ß*	**0.310**	−0.077	0.137	**0.371**	0.121	0.138
*p* value	**0.009**	0.540	0.357	**0.001**	0.287	0.225
Psychological health						
*R* ^2^	0.063	0.015	0.031	0.245	0.009	0.010
Standardised *ß*	**0.259**	0.096	0.144	**0.236**	0.030	0.045
*p* value	**0.038**	0.456	0.354	**0.043**	0.799	0.702
Social relationships						
*R* ^2^	0.006	0.003	0.008	0.021	0.014	0.003
Standardised *ß*	−0.041	0.014	−0.022	0.137	0.107	0.004
*p* value	0.742	0.911	0.884	0.234	0.354	0.975
Environment						
*R* ^2^	0.049	0.028	0.025	0.088	0.047	0.030
Standardised *ß*	0.160	0.026	0.087	**0.253**	0.143	0.048
*p* value	0.187	0.836	0.568	**0.024**	0.207	0.676
WHOQOL-OLD						
Sensory ability						
* R* ^2^	0.120	0.116	0.059	0.116	0.138	0.117
Standardised *ß*	0.071	−0.022	−0.017	0.006	−0.154	−0.036
*p* value	0.542	0.852	0.908	0.955	0.155	0.740
Autonomy						
*R* ^2^	0.099	0.095	0.002	0.123	0.084	0.055
Standardised *ß*	**0.244**	0.237	0.088	**0.276**	0.184	−0.063
*p* value	**0.049**	0.058	0.589	**0.015**	0.106	0.583
Past, present and future activities						
* R* ^2^	0.014	0.011	0.004	0.124	0.021	0.010
Standardised *ß*	0.090	−0.058	−0.009	0.090	−0.117	0.042
*p* value	0.508	0.674	0.956	0.584	0.344	0.741
Social participation						
* R* ^2^	0.202	0.018	0.018	0.267	0.150	0.087
Standardised *ß*	**0.478**	0.088	0.109	**0.518**	**0.381**	**0.282**
*p* value	**<0.001**	0.484	0.466	**<0.001**	**0.001**	**0.013**

*n* = 83

Significant results are shown in bold

According to the multiple linear regression analysis (Table 4), DPA, handgrip strength and balance skills together explained 31 % of the variance in ‘overall QoL’. Furthermore, balance skills alone explained 20 % of the QoL domain of ‘physical health’. DPA, handgrip strength and balance skills were independent indicators for the QoL domain of ‘autonomy’ (*R* ^2^ = 0.247), whereas balance was the strongest indicator. Moreover, DPA and balance skills together explained 36 % of the variance in the QoL domain of ‘social participation’, and balance again showed the strongest association.

**Table 4:** Model—multiple linear regression model including all independent variables (e.g. handgrip strength) and one QoL domain (dependent variable), adjusted for sex and age

	*R* ^2^; *p* value	Included independent variables^a^	Standardised	*p* value
WHOQOL-BREF				
Overall QoL	0.309; *p* < 0.001	Daily physical activity	0.274	0.027
		Handgrip strength	0.345	0.004
		Balance skills	0.180	0.125
Physical health	0.200; *p* = 0.001	Balance skills	0.389	0.001
Psychological health	0.073; *p* = 0.160	Balance skills	0.246	0.044
Social relationships	0.002; *p* = 0.940			
Environment	0.052; *p* = 0.284	Balance skills	0.205	0.087
WHOQOL-OLD				
Sensory ability	0.059; *p* = 0.114			
Autonomy	0.247; *p* = 0.004	Daily physical activity	0.192	0.152
		Handgrip strength	0.285	0.029
		Balance skills	0.333	0.014
Past, present and future activities	0.004; *p* = 0.895			
Social participation	0.356; *p* < 0.001	Daily physical activity	0.299	0.012
		Balance skills	0.418	<0.001

*n* = 83

^a^Only variables with a *p* value threshold of 0.20 were included

## Discussion

The main findings indicated that there was no association between skeletal muscle mass and QoL, whereas balance skills, DPA and handgrip strength were associated with QoL. Furthermore, balance was the factor most strongly associated with the QoL domains of ‘physical health’, ‘autonomy’ and ‘social participation’.

Before discussing the associations, it ought to be mentioned that when compared to previous trials, our participants scored similar values in the QoL domains, except for ‘social relationships’, ‘environment’ and ‘physical health’ [30, 31]. The higher scores in the ‘social relationship’ domain might be explained by the fact that we excluded the question ‘How satisfied are you with your sex life?’ Higher scores in the ‘environment’ domain might be explained by the different environmental circumstances of the countries. Lower scores in the ‘physical health’ domain might be traced back to the fact that we only included prefrail and frail persons, i.e. persons with defined physical limitations.

The correlation between handgrip strength and QoL is in accordance with other studies [6, 32, 33]. As handgrip strength is an overall measurement of body strength in older adults [34, 35], our results indicate that muscle strength is an important factor for QoL, whereas muscle mass is not. Hence, muscle quality and factors such as muscle composition, neural activation, metabolism and fibrosis might be relevant [13–15]. Our findings also revealed that balance was the variable most strongly associated with the various QoL domains. An association between balance and poorer QoL was also described by Gobbens et al. [7]. This relationship might be due to the fact that balance is the most important requirement in daily life [36], and problems in maintaining balance lead to a restriction of activities due to the fear of falling [37]. In this context, it is noteworthy that muscle strength and muscle mass are important biomechanical requirements for maintaining balance [38]. However, in our sample, neither muscle strength nor muscle mass was found to be associated with balance skills. The nonsignificant correlation is comparable to the findings of Visser et al. [34] and a British study [39]. A reduction in the association between muscle strength and balance over the lifespan has also been confirmed in the current literature [40], with a change in the neuromuscular components being identified as the underlying reason [40]. Misic et al. [41] showed that muscle quality and not muscle mass was the strongest independent factor for balance in older adults. However, apart from muscle quality, limitations in the sensory system (visual, vestibular, proprioceptive, tactile somatosensory), cognitive impairments and orthopaedic problems also influence balance [36, 38]. Hence, the data indicate that it is not muscle mass, but rather factors such as muscle quality, constraints in the sensory system and orthopaedic problems that are closely linked to QoL in prefrail and frail persons. Further research on this assumption is needed.

As previous studies have showed, ‘social participation’ and contact with neighbours are important factors for the well-being and mental health of older persons [42, 43]. As the recent study of Etman et al. [44] showed that limited ‘social participation’ is associated with further worsening of frailty symptoms, this QoL domain is of special interest. Our data showed that individuals with better DPA and balance skills have a better QoL in ‘social participation’, indicating that balance and DPA should be kept as high as possible. However, it could also be the other way around: QoL in the ‘social participation’ domain should be increased to enhance balance and DPA. Apart from this, the fact that good balance is an essential precondition for leaving the house and for participating in social activities might be the reason for the close association. The same considerations might also apply to the QoL domain of ‘autonomy’.

A major strength of our study was the inclusion of very old community-dwelling prefrail and frail subjects. We used reliable and valid measurements to assess variables such as muscle mass, DPA and QoL. One limitation to the study design was that a temporal and causal link between independent variables and QoL could not be proven. The small sample size was another limitation. Nevertheless, we were able to detect the effects of the physical training and nutritional intervention carried out by the trained lay volunteers. However, the internal consistency was lower than in other validation studies [45, 46]. Hence, an acceptable internal consistency of >0.70 [47] was only achieved in the ‘sensory ability’ domain. However, the domain scores were sufficient for the study purpose, as the correlation between the items in each domain was adequate. Nevertheless, these questionnaires should be validated for prefrail and frail persons in further research. Moreover, ASMM was calculated based on the results of the bioelectrical impedance analysis using the validated formula of Sergi et al. [24], who validated the ASMM calculation for individuals with a mean age of 71.4 years (SD: 5.4) without chronic comorbidities. Due to this fact, this formula might not be directly comparable to our study participants since our population was both older and had chronic comorbidities.

## Conclusion

As skeletal muscle mass was neither associated with ‘overall QoL’ nor with any QoL domain, skeletal muscle mass can be considered as not playing a role in the QoL context of prefrail and frail older persons. However, balance skills and DPA are relevant factors. These parameters were particularly associated with the QoL domains of ‘social participation’ and ‘autonomy’. However, we do not know whether low balance skills and low DPA are the cause or the consequence of low QoL.
